# Protein Data Bank Japan: Computational Resources for Analysis of Protein Structures^✩^

**DOI:** 10.1016/j.jmb.2025.169013

**Published:** 2025-02-15

**Authors:** Gert-Jan Bekker, Chioko Nagao, Matsuyuki Shirota, Tsukasa Nakamura, Toshiaki Katayama, Daisuke Kihara, Kengo Kinoshita, Genji Kurisu

**Affiliations:** 1 - Institute for Protein Research, Osaka University, 3-2, Yamadaoka, Suita, Osaka 565-0871, Japan; 2 - Tohoku Medical Megabank Organization, Tohoku University, Sendai, Miyagi 980-8573, Japan; 3 - Advanced Research Center for Innovations in Next-Generation Medicine, Tohoku University, Sendai, Miyagi 980-8573, Japan; 4 - Graduate School of Information Sciences, Tohoku University, Sendai, Miyagi 980-8579, Japan; 5 - Department of Biological Sciences, Purdue University, West Lafayette, IN 47907, USA; 6 - Structural Biology Research Center, Institute of Material Structure Science, High Energy Accelerator Research Organization, 1-1 Oho, Tsukuba, Ibaraki 305-0801 Japan; 7 - Database Center for Life Science, Joint Support-Center for Data Science Research, Research Organization of Information and Systems, Kashiwa, Chiba 277-0871, Japan; 8 - Department of Computer Science, Purdue University, West Lafayette, IN 47907, USA; 9 - Protein Research Foundation, Ina 4-1-2, Minoh, Osaka 562-8686, Japan

**Keywords:** PDB, data archives, UniProt, protein structure, sequence analyses

## Abstract

Protein Data Bank Japan (PDBj, https://pdbj.org/) is the Asian hub of three-dimensional macromolecular structure data, and a founding member of the worldwide Protein Data Bank. We have accepted, processed, and distributed experimentally determined biological macromolecular structures for over two decades. Although we collaborate with RCSB PDB and BMRB in the United States, PDBe and EMDB in Europe and recently PDBc in China for our data-in activities, we have developed our own unique services and tools for searching, exploring, visualizing, and analyzing protein structures. We have also developed novel archives for computational data and raw crystal diffraction images. Recently, we introduced the Sequence Navigator Pro service to explore proteins using experimental and computational approaches, which enables experimental structural biologists to increase their insight to help them to design their experimental studies more efficiently. In addition, we also introduced a new UniProt-integrated portal to provide users with a quick overview of their target protein and it shows a recommended structure and integrates data from various internal and external resources. With these new additions, we have enhanced our service portfolio to benefit both experimental as computational structural biologists in their search to interpret protein structures, their dynamics and function.

## Introduction

The three-dimensional structural data of biological macromolecules are collaboratively maintained by the worldwide Protein Data Bank (wwPDB) partnership. Protein Data Bank Japan (PDBj, https://pdbj.org), has accepted and processed the 3D structure data of biological macromolecules from Asia and distributed the globally collected data since 2000.^1^ In total, roughly 23% of all PDB entries had been processed by PDBj by the end of 2023. Since our founding, PDBj has developed various original services, which are listed in Table 1. Here, we will describe updates to our original services and archives, as well as introduce several newly developed services to assist both experimentalists and structural data users alike.

## Overview of archives maintained by PDBj

PDBj maintains three wwPDB core archives (Protein Data Bank: PDB, Electron Microscopy Data Bank: EMDB, and Biological Magnetic Resonance Data Bank: BMRB) under the wwPDB partnership in collaboration with the other wwPDB members,^2^ while we also maintain uniquely developed archives. The PDB data we co-maintain together with RCSB PDB in the USA and PDBe in Europe. Together with the EMDB team at EMBL-EBI in Europe, we co-maintain the EMDB archive for experimental 3DEM maps. For NMR data, PDBj collaborates with the BMRB team in the USA to maintain the BMRB archive as part of our BMRBj activities.^3^ Deposition to these wwPDB core archives is handled via the OneDep system, which is shared among all wwPDB partners. Here, PDBj manages the Asian depositions of experimental data submitted to the archives, corresponding to approximately 27% of the worldwide depositions over the past 5 years. Since 2018, we have also maintained a mirror of EMPIAR (the Electron Microscopy Public Image Archive: https://www.ebi.ac.uk/empiar/), in collaboration with the team at EMBL-EBI.^4^ Our mirror site focuses on the PDB-related EMPIAR entries, and our mirror site (EMPIAR-PDBj) is slightly different from the master archive at EMBL-EBI. In addition, to assist with depositions from Asia, we also broker the deposition of entries to EMPIAR, where we also accept HDD submissions via postal mail, where we then upload the data to EMBL-EBI. We have also developed two novel archives; BSM-Arc (the Biological Structural Model Archive) for computational data and XRDa (the Xtal Raw Data Archive) for experimental diffraction images. Thereby, PDBj collects both raw experimental data via BMRBj, EMPIAR-PDBj and XRDa, experimental structural models via OneDep, and computational structural models and trajectories via BSM-Arc, and is therefore the only wwPDB partner that collects raw data for all experimental types and from computational sources.

PDBj also maintains several secondary databases. These secondary databases use data from the primary PDB archive and use computational methods to derive additional insights, which are stored in their respective archives. The Promode Elastic service provides information with respect to predicted dynamics of a PDB entry (or specific chains), calculated via Normal Mode Analysis (NMA), and is updated on a weekly basis.^5^ The eF-site service is a database containing the calculated electrostatic potentials mapped on the molecular surfaces of functional sites.^6^ Finally, the Dynamics DB is a service that provides stability and dynamics of proteins calculated via molecular dynamics (MD) simulations.

## PDBj tools for data-in and original archives

**Figure 1.**
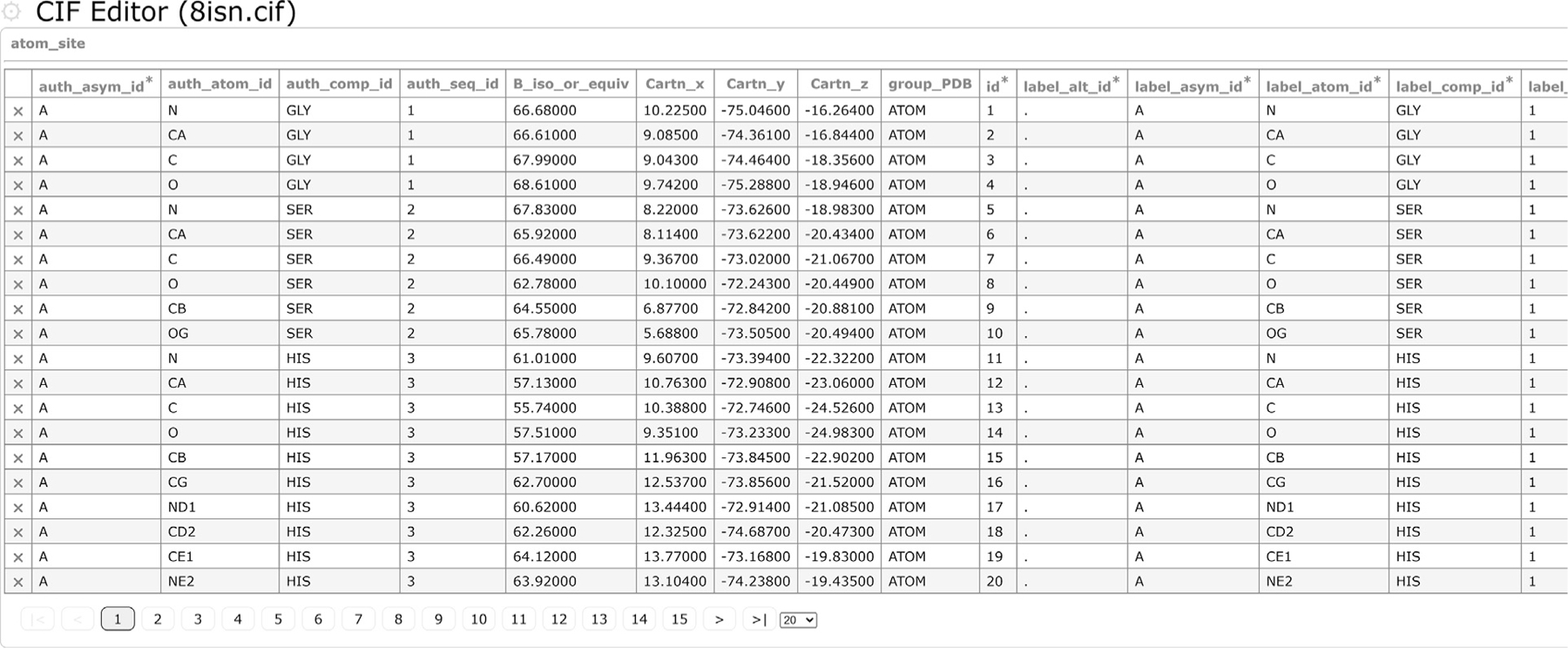
CIF Editor. Example of loading in an PDBx/mmCIF file. Here, an already released PDB entry was used, in particular PDB ID 8ISN. Files (including gzipped ones) can be dropped in directly or can be loaded in by clicking on the main menu icon (⚙), and then selecting “Open mmCIF file”. Via this same menu, the file can later be saved. Each category is shown in a separate panel, with the contents listed in a table. Additional categories can be shown by selecting them via the “Toggle tables” option in the main menu. In the first row of the table, the data names are listed, and additional columns can be toggled via the “Toggle columns” option in the category menu, which can be shown by clicking on the category name above the table. The data name menu can be shown by clicking on a data name, and can be used to search for data, filter data or perform batch operations. Clicking on the “X” symbol in the first column deletes the row, while clicking on any of the data fields in the table converts it into an input box (of different types, depending on the data type defined in the mmCIF editor), to modify the content. On deselecting the input box, the content is checked against the dictionary and stored in-memory. Below the table are pagination controls, to view different parts of the data table. In case a user-supplied file is loaded, validation is performed on the data against the dictionary and a popup will be shown with any incompatible data, which will also be marked by red in the corresponding category data tables, and upon saving, the data is once more validated. Finally, the raw editor is accessible via the category menu and after completing the manual modifications, the raw data can be merged into the entry again via the “Validate CIF & update” option in the main menu when in the raw editing mode, which also performs a validation.

To help depositors grow accustomed to the mmCIF format, we have created an mmCIF editor (https://pdbj.org/cif-editor/),^4,7^ and due to its generalized implementation, the CIF Editor is also used by our archives BSM-Arc and XRDa to register and modify metadata during deposition. The CIF Editor runs inside a web browser and does not require any installation, ensuring that users will always use the most recent version, without having to wait to install an update before every use. Although the editor can load mmCIF files directly from the web, it can also load user-supplied files, by drag-and-dropping the files directly into the page. The data is fully processed within the user’s local web browser, and no data is sent to any of our servers. Upon loading, it will try to determine the correct dictionary to use and otherwise will prompt the user for the correct dictionary, which will then be used to validate the contents of the supplied mmCIF file, as well as provide on-the fly validation as the user modifies the file. The default editing mode is the UI based mode, where the categories are loaded into tables and users can modify individual data-items or perform batch operations. The batch operations can also be combined with filter operations, e.g., to first filter by chain (atom_site.label_asym_id), and then renumber the residues (atom_site.label_seq_id) via a batch operation. Alternatively, there is a manual mode that allows users to directly edit the raw mmCIF data. After switching to the raw editing mode, users can freely edit the mmCIF data manually, after which the editor will re-assimilate the modified content while validating it against the mmCIF dictionary. Finally, their file can be saved to their local disk as either an mmCIF or mmJSON file. Figure 1 provides an overview of the interface and a description of where to find each of the functions.

In 2018, we developed a new archive for computational data, the Biological Structure Model Archive (BSM-Arc)^8^ (https://bsma.pdbj.org/). The archive has since published 54 entries, encompassing 6.85 TB of data. Depositors can login using their ORCID ID to create and manage entries, where multiple depositors can simultaneously manage the same entry once they have been registered within the entry by the initial depositor. Metadata and additional descriptions can be added via a web interface (Figure S1), while files can also be uploaded via this web interface. Here, uploading of files is accelerated, by uploading multiple small files in parallel, and larger files by uploading multiple chunks in parallel. Alternatively, we also provide an option to upload files via the RSYNC protocol, which can then be imported into the BSM-Arc entry. Each entry is also assigned a DOI, which can be used to cite the BSM-Arc entry. Data published in BSM-Arc can also be downloaded via our data archive, which includes data from all our data-out sources. Via the web interface, individual files can be downloaded, while a zipped file of the full entry can also be downloaded. For known file formats, a web viewer is assigned, so that double clicking on any file in the file manager will show the contents of the file. For molecular structures (e.g., PDB files), our molecular viewer, Molmil, is used to visualize them, and if Molmil scripts are used, complex, pre-programmed visualizations or animations can be applied.

At the beginning of the COVID-19 pandemic, we launched a new archive called the Xtal Raw Data Archive (XRDa, https://xrda.pdbj.org/). Both the USA and Europe already had their own archives with IRRMC^9^ and SBDB,^10^ but no such archive existed yet in Asia. The archive has published 181 entries, encompassing 9.8 TB of data. We welcome depositions of raw diffraction images, both macro-molecular crystallography and chemical crystallography, collected using either X-ray, electron, or neutron diffraction. Like BSM-Arc, depositors can login using their ORCID ID to make depositions to XRDa. Furthermore, XRDa is loosely linked with the wwPDB’s OneDep system, and has access to the list of depositors (in particular, their ORCID IDs) and the PDB entries that have been submitted via OneDep. Therefore, after submitting their data to OneDep, submitting diffraction data to XRDa is trivial, as their newly submitted PDB entry shows up in the XRDa PDB entry list, ready to accept data. For older entries that pre-date ORCID registration in OneDep, we can manually add links between depositors’ ORCID ID and the old PDB entries, so please do not hesitate to contact us if you want to submit data for older entries. It is also possible to publish diffraction data unrelated to PDB entries, or to link a PDB entry post release, e.g., if you want the XRDa entry to be published before the PDB entry. In case an XRDa entry is linked with a PDB entry, the XRDa entry will be automatically co-published with the PDB entry. Therefore, if the PDB entry has an HPUB status, the XRDa entry will be published at the exact same time as the PDB entry, while if the PDB entry has already been published, the XRDa entry will become immediately available after completing the submission process at XRDa. Since the interface of XRDa is based on that of BSM-Arc, many of the same functionalities are available, including managing meta-data and uploading files, either via the web interface or via the RSYNC protocol (Figure S2). For the data-out side, individual files can be downloaded and visualized, as well as the full entry in a ZIP archive. The data is also available from our data archive via RSYNC. Finally, each entry is also assigned a DOI number, which can be used to cite the XRDa entry.

## PDBj tools and services for data-out

In addition to the primary and secondary archives that we maintain, we have also developed several tools and services.^11,12,4^ Table 1 provides an overview of the available services and their links. To explore the PDB archive, we have developed the PDBj Mine service. Central to the PDBj Mine service is the Mine 2 relational database (RDB), which contains the meta-data of all PDB entries, as well as the metadata for the chemical component dictionary (chem_comp), PRD/BIRD (Biologically Interesting Molecule Reference Dictionary) and PDB validation reports (VRPT). For Cryo-EM data, the EMDB data, whose metadata are now also distributed as PDBx/mmCIF formatted files by wwPDB members as well as PDBx/mmJSON by PDBj, and EMPIAR data are also queryable. In addition, metadata from various sources and other calculations are also included, such as file information, release statistics, obsolete entry information and inter-molecular contact pairs. Since all this data is available within a single RDB, complex queries can be crafted to perform very precise searches, and/or extract data for many entries with a single query. PostgreSQL dump files of the RDB are available from our data archive, while we also provide software to automatically maintain an up-to-date local copy of the RDB (https://git-lab.com/pdbjapan/mine2updater). We provide multiple interfaces for the PDBj Mine service. First is the quick search interface, which can be found in the header of our website and is a basic keyword search, but additional filtering for common queries can be applied. Second is our advanced search by using an RDB search interface to directly search all aspects of the RDB, which can either be used via a graphical interface or via a simple SQL interface. Although these direct RDB search interfaces are much more powerful, knowledge of both the data structure (as described in the mmCIF dictionary), as well as SQL query syntax is required. Here, the graphical interface only requires users to have knowledge of the mmCIF data structure, although novice users could use the included help interface to search for data categories/tables instead. Therefore, by using the graphical interface, filtering using any data part of the PDB can be performed without requiring any SQL knowledge, but for more complex queries beyond filtering, such as alternate data extraction or for complex filtering, the text-based SQL-query based approach is still more powerful. These search interfaces are also query able via our REST services as described on our help page https://pdbj.org/help/rest-interface.

To explore individual PDB entries, we provide the Mine web interface, which describes and explains various aspects of released PDB structures. Our molecular viewer, Molmil,^13^ is used to provide various visualizations of the structure, including the asymmetric unit, biological unit and electron density maps, if available. Recently, we introduced a 2D interactive representation of the structure topology, which is a simplified representation of the interactions between the various molecules in the PDB entry. For EM entries, we also provide links to the DAQ-Score Database, which provides a quality assessment of EM-derived structures.^14,15^ We also expanded the interface describing the experimental details of crystallography derived structures (Figure S3). Additional information describing the experimental procedure, the refinement procedure and the data characteristics are now shown. Recently, structures derived via integrated/hybrid methods were added to the PDB archive as the PDB-IHM (Integrated/Hybrid Methods), which are also visualized via our Mine interface in a comparable manner to the regular PDB entries. With Chemie, we provide a search interface to the chemical compound dictionary data part of the PDB. A search interface is provided to search and filter the compound library like PDBj Mine for PDB entries, and individual entry pages are also made available, providing information about the chemical structure, visualization using Molmil and links to PDB entries that contain the chemical compound, with finally also a similar interface also provided for PRD/BIRD (Biologically Interesting Molecule Reference Dictionary) entries. All entries can be searched for using our quick search interface, or via our advanced search methods, and links between related entries across databases and archives are provided.

Molmil (https://pdbj.org/molmil2/) is a WebGL-based molecular viewer that we have been developing since 2013 and is used by PDBj for various services.^11,12,13,4^ Molmil can also be used as a standalone viewer to load user-provided structures and MD trajectory files, without requiring any installation. We also provide an installable version called molmil-app is also available to enable shell-based loading and headless processing (our images of PDB structures are generated in this manner). During the development of Molmil, we also developed a new format called PDBx/mmJSON, which uses the same definitions (and dictionary) as PDBx/mmCIF, but encodes the data in a JSON format, which can be read by any modern programming language, without requiring a custom mmCIF parser, while being on average about 33% smaller than the corresponding mmCIF file. Finally, we provide a REST service that can produce data in mmJSON format for selected categories for an entry.

We have also developed several services to explore and analyze 3DEM structures. In 2007, we started our EM Navigator service,^16^ a website to explore 3DEM data in the EMDB and PDB. The EM Navigator service produced short movies that stored representations of the 3DEM data from different orientations to help users to visualize and understand the models. The Omokage service was developed as a shape similarity search service for 3D structures of macromolecules that compares the overall shape between registered structures or a user-submitted one.^17^ The gmfit service also works on EM data and can be used to quickly fit 3D objects (either structures or density maps) using Gaussian mixture models.^18^

We have also developed several services to perform sequence-based analyses. To enable sequence homology searches within the PDB, we provide the Sequence Navigator service, which enables searching the PDB for homologous structures given a query sequence. Similarly for existing PDB entries, our Sequence Neighbor service enables searching for homologous structures and visualizing their superposed structures using Molmil. The CRNPRED service can be used to predict characteristics of a protein such as secondary structure, contact numbers and residue-wise contact orders from the amino acid sequence.^19^ While CRNPRED uses the amino acid sequence to predict structural properties, our HOMCOS service can be used to model the quaternary structure of proteins based on homology modelling.^20^ In addition, it can also be used to search for potential binding compounds given an amino acid sequence, or a set of binding proteins given a compound.

## Sequence exploration for data-in

**Figure 2.**
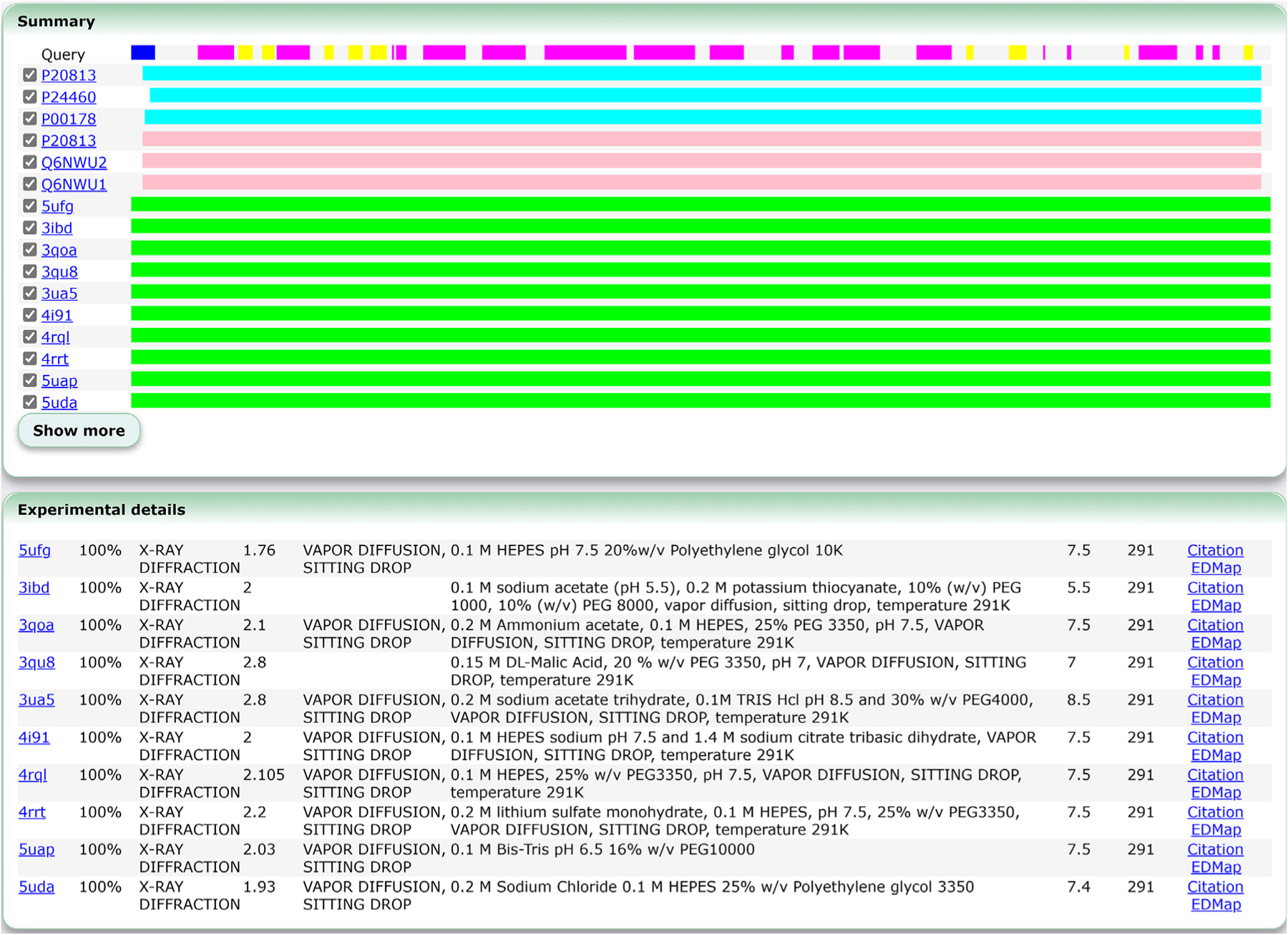
Example of the new Sequence Navigator Pro service. In the top panel, the summary panel of the results are shown. Here, a list of the ranked hits is shown. Initially the top three from SwissProt, top three from AFDB and top ten from the PDB are shown. Clicking on the “Show more” button shows the additional hits. Selecting the hits by checking the checkbox selects the hits for further analysis. In the bottom panel, the experimental details of the selected PDB homology matches are shown. Experimental details for the selected PDB entries in the summary panel are listed here. Query hit rate, experimental method, resolution in A, experimental conditions, pH and temperature in K are shown. Additional contents are shown in Figure S4.

We recently introduced the Sequence Navigator Pro service (https://pdbj.org/seqnavipro). Although we have long provided the original Sequence Navigator service, which is a homology-based PDB entry search service, it is limited in terms of analyzing the detailed characteristics of a query sequence and the homologues discovered. Although the Sequence Navigator Pro service still performs a homology search against the PDB using BLAST,^21^ it also analyzes the sequence in several other ways and packages up the results in a more usable manner. First, it also performs a homology search against the SwissProt KB sequence archive and the AlphaFold DB (AFDB, https://alphafold.ebi.ac.uk/) computational structure archive. This enables users to search for a structure (either experimental or computationally derived) if they only have their sequence available. The service then lists the top-ranking matches in terms of their sequence coverage (Figure 2). Hits can furthermore be toggled, which are then used for further analysis. Next, a panel that describes the experimental details of the toggled PDB hits is shown, for a quick overview of the characteristics of the matching structures, experimental procedures as well as any chemicals used during crystallization (Figure 2). In addition, a link to the primary citation of the PDB entry is shown if available. Also shown is a link to our EDMap service to view the electron density maps using our molecular viewer Molmil in case structure factors of MX entries were deposited. The experimental details panel provides detailed information regarding the experiments previously performed for similar structures and should provide some insight into the experimental parameters that should lead to successful elucidation of the structure. We also perform some predictions based on the sequence, where we predict the secondary structure using s4pred,^22^ predict disordered regions using flDPnn,^23^ as well as estimates the hydropathy based on the sequence (Figure S4). Finally, we provide an effortless way to then perform keyword search against the linked literature (PubMed) of the discovered homologous PDB and SwissProt entries (Figure S4). This can provide additional information and context related to the sequence, while limiting the results to only closely matching sequences, to reduce search time.

## Dynamics DB

We have also created a new archive of protein stability derived from an analysis of MD simulations(https://bsma.pdbj.org/dynamicsdb/, Figure S6, see also Section S1 for a detailed description of the MD simulations). Contact matrix analysis of high temperature MD simulations were previously shown to show a good correlation between the experimental Tm values,^24^ as well as for binding simulations.^25^ Here, this analysis was performed on a subset of the PDB (9562 entries), where the data was subsequently stored in the newly created archive. The secondary database can provide insights into the local stability and interactions within proteins to provide clues as to the effect mutations might have on internal interactions and stability. We also developed an interface to visualize the per-residue stability, where in the top half, a graph that shows the stability (represented by the R-value) of each of the residues is shown (black) along with the contact number for each residue (blue). The R-value is a measure of the stability of the contacts measured during the MD simulations with respect to a representative structure, which was also calculated from the MD simulations and corresponds to the structure whose contact matrix is the closest to the average contact matrix. In the bottom panel, Molmil is used to visualize the stability of the structure. The representative structure is shown, which is colored (cartoon and carbons) based on the stability, where blue corresponds to stable residues (R-value = 1.0) and red to unstable residues (R-value = 0.0). From the PDBj Mine page, there are links available to the Dynamics DB page for the entries that have been analyzed.

## Sequence exploration for data-out

To make it easier for users to select a structure for a given target protein, we set out to develop a new sequence-oriented portal service. The largest sequence database of protein sequences is the UniProt Knowledgebase (KB), which comprises of the UniProtKB/Swiss-Prot component for a reviewed and manually annotated protein dataset, and the UniProtKB/Trembl component for an unreviewed and largely computationally annotated protein dataset.^26^ Therefore, we constructed our new portal page using the UniProt IDs to link them with the structures in the PDB archive. In addition to the UniProt protein data, we have also integrated two genomic databases; Japanese Multi-Omics Reference Panel (jMorp) and Medical Genomics Japan Variant Database (MGeND). The jMorp database is a secondary database produced from the analysis of data registered in the Tohoku Medical Megabank (TMM)^27^ that provides a statistical overview of the genetic diversity of the TMM Cohort. MGeND^28^ is a curated database of genetic mutations that are involved in clinical observations. We have mapped the genomic data from jMorp and MGeND onto the corresponding UniProt entries, which subsequently allows them to be mapped to the corresponding PDB structures and thereby integrated both resources into our new service.^29^

**Figure 3.**
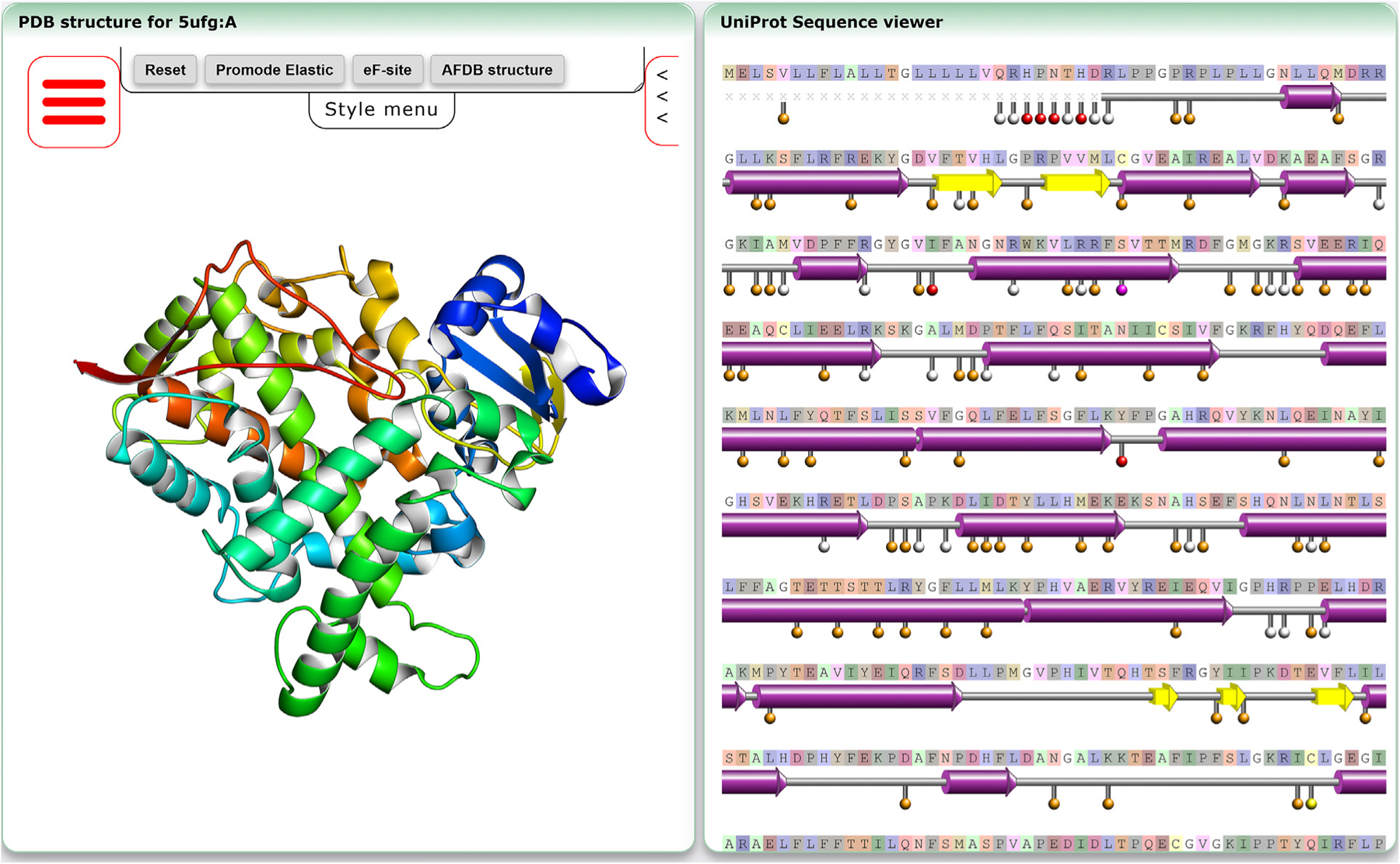
Example of new UniProt portal entry showing the structure & sequence visualization. As an example, the entry page corresponding to P20813 is shown (https://pdbj.org/uniprot/P20813). In the left panel, the representative structure is shown, colored in a blue-red gradient along the N-to C-terminus. For an AFDB template, the structure is instead colored by its quality score. For PDB templates, if additional data is available from our secondary archives Promode-Elastic, eF-site and Dynamics DB, buttons to toggle the visualization of these resources are shown. In addition, if DAQ scores are available, or an AFDB structure is available, buttons to toggle visualization for these resources are also shown. In the right panel, the sequence of the UniProt entry is shown, with the secondary structure taken from the PDB structure or AFDB structure (depending on which is shown in the left panel). Residues that are not part of the PDB entry are indicated by grey crosses, while residues that are part of the PDB entry (i.e., expressed), but were not observed, are shown as red crosses. In addition, various sites from the UniProt entry or from external resources are indicated along the sequence. Here, orange circles correspond to mutation sites, mutations in the PDB structure correspond to red circles, sites involved in covalent bonds correspond to green circles, interaction sites correspond to blue circles, glycosylation sites correspond to cyan circles, binding sites correspond to yellow circles, post-translational modification sites correspond to magenta circles and mixed sites correspond to white circles.

**Figure 4.**
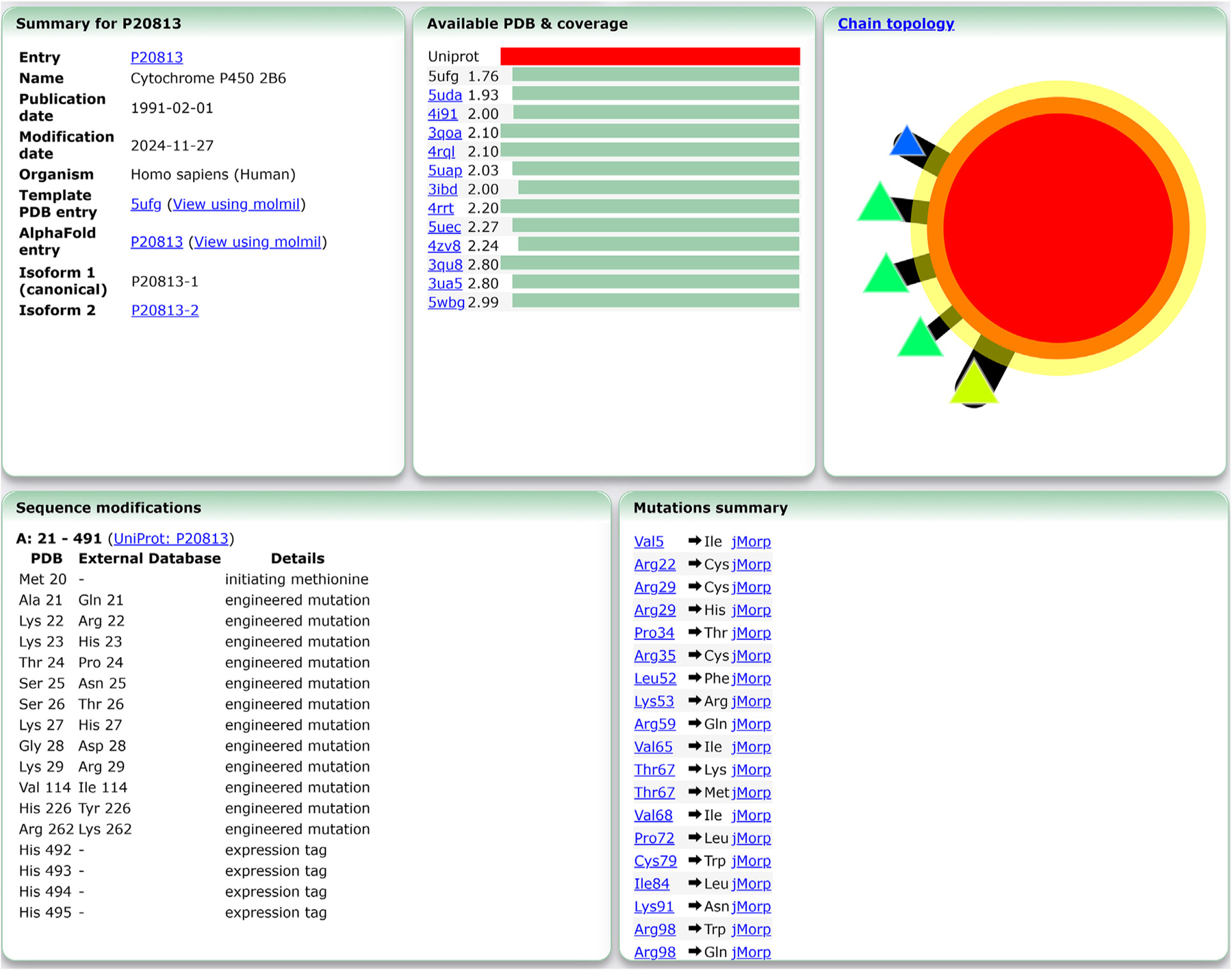
Example of new UniProt portal entry showing entry, structure and mutation information. In the top row, the entry summary, PDB coverage and chain topology representation are shown. The summary panel on the left side shows basic information regarding the entry, as well as links to either the PDBj Mine page or the AFDB page of the template structure. If any isoforms are present, links to the UniProt portal entries of these isoforms are also provided. The center panel lists all available PDB structures, ordered by their resolution and sequence coverage relative to the UniProt sequence. The chain topology panel on the right shows a simplified rendering of the template PDB structure, with the chains corresponding to the UniProt entry indicated by a yellow halo. In the bottom row, sequence modifications and mutations are shown. If any modifications in the template PDB structure exist with respect to the UniProt sequence, the Sequence modifications panel is shown (left panel). If there are any mutations recorded in either jMorp or MGeND, these are shown in the Mutations summary panel (right panel). If in either case no such sites are present, the corresponding panel is not shown.

The new service integrates both sequence data from UniProt, as well as structural data from the PDB. Figures 3 and 4 show an example of the portal page for entry P20813 (https://pdbj.org/uniprot/P20813). In Figure 3, in the right corner, the UniProt sequence is shown using our sequence viewer applet.^11,12,4^ For each entry, a template PDB chain is shown from the PDB in the left panel, visualized using Molmil. However, if no structure from the PDB is available, a computational structure obtained from AFDB is shown instead (if available). In case a PDB structure was used, the secondary structure assignments from the PDB structure are used and shown in the sequence viewer panel. Otherwise, secondary structure assignments calculated by Molmil from the AFDB structure are shown. Also shown along the sequence are known ligand binding and glycosylation sites recorded in the UniProt entry, as well as the locations of the genomic variation recorded in the UniProt entry or obtained from jMorp and MGeND. Clicking on any of the residues within the sequence viewer will cause Molmil to jump to and show that residue, if available in the structure. For PDB templates, if there is a compatible entry available from one of our secondary structure archives Promode-Elastic, Dynamics DB, or eF-site, their data can be directly visualized onto the template PDB structure. In addition, the AlphaFold structure (if available) can also be co-visualized, as well as coloring the structure based on the DAQ-Score, if available.

In addition, several other panels that describe the entry, available structures and mutants are shown in Figure 4. The UniProt summary panel shows a summary of the entry, including links to the AFDB structure page, if available, and it lists any links for isoform entry pages, if available (Figure 4). The available PDB & Coverage panel lists any PDB entries that are available from the PDB for this UniProt entry with their resolution, as well as provides a visual indicator to how much of the UniProt sequence is covered by each of the PDB entries. Using this information, i.e., the resolution of the PDB entries and their sequence coverage, a representative PDB structure is chosen as the template structure, although other structures, including the AFDB structure, can also be used as a template structure. A simplified intermolecular representation of the topology, which is also shown on the Mine PDB entry page, is also shown, with the chains corresponding to the UniProt entry indicated by a yellow halo. Changes to the PDB structure with respect to the sequence recorded in the UniProt entry are also shown, along with a panel that lists the mutations obtained from jMorp or MGeND. Finally, the PDB structure summary panel and the structure validation panel are shown for the selected PDB template structure (if available).

## Conclusion

PDBj has developed and updated several original tools to help users to find/access/interoperate/reuse the PDB/BMRB/EMDB entries. In addition, PDBj has developed several novel archives for experimental and computationally derived data, as well as tools to manage and annotate the data deposited to these archives or to the PDB. This data has all been integrated into the PDBj website, which thereby provides access to a vast amount of data. To sift through this amount of data we have created several low and mid-level tools. However, since the PDB consists of many structures, it can sometimes be challenging to find the most suitable structure for a given protein. Our new sequence-oriented services will help users identify the most suitable structure for their protein, be it an experimental structure or a computationally derived one. In addition, integration with external genomic resources will provide insight into the genomic and potentially structural variability of the proteins. Finally, integration with our secondary archives provides additional information with respect to the properties of the proteins.

## Supplementary Material

Supplementary file

Appendix A. Supplementary material

jmb24_pdbj_SI.pdf: Section S1 with detailed description for the preparation, molecular dynamics simulations and analysis performed for the construction of the Dynamics DB. Figure S1 showing the deposition interface of BSM-Arc, Figure S2 showing the deposition interface of XRDa, Figure S3 showing the new experimental details for crystallography-derived structures, Figure S4 and S5 shows the additional results provided by our new Sequence Navigator Pro service and Figure S6 showing an example of a Dynamics DB entry. Supplementary material to this article can be found online at https://doi.org/10.1016/j.jmb.2025.169013.

## Figures and Tables

**Table 1:** PDBj services and tools with corresponding URLs.

Service	URL
Search PDB (PDBj Mine)	pdbj.org/search/pdb-filter
Chemie search	pdbj.org/chemie-search
Search BMRB	bmrbj.pdbj.org
Sequence-Navigator	pdbj.org/seq-navi
EM Navigator	pdbj.org/emnavi
Omokage search	pdbj.org/omokage
wwPDB/RDF	rdf.wwpdb.org
jV: Graphic Viewer	pdbj.org/jv/
Molmil: WebGL Molecular Viewer	pdbj.org/molmil2/
Yorodumi	pdbj.org/emnavi/
NMRToolBox	bmrbj.pdbj.org/en/nmr_tool_box.html
gmfit	pdbj.org/gmfit/
CRNPRED	pdbj.org/crnpred/
HOMCOS	homcos.pdbj.org
eF-site	pdbj.org/eF-site/
eF-seek	pdbj.org/eF-seek/
eF-surf	pdbj.org/eF-surf/
ProMode Elastic	pdbj.org/promode-elastic
Molecule of the Month	numon.pdbj.org/mom/
Games	numon.pdbj.org/games/
Paper models	numon.pdbj.org/papermodel/
OneDep (Deposition to PDB, EMDB or BMRB)	deposit-pdbj.wwpdb.org/deposition
Format Conversion	mmcif.pdbj.org/converter/
PDBx/mmCIF editor	pdbj.org/cif-editor/
EMPIAR-PDBj	empiar.pdbj.org
BSM-Arc	bsma.pdbj.org
XRDa	xrda.pdbj.org
UniProt portal	pdbj.org/uniprot/
Sequence Navigator Pro	pdbj.org/seqnavipro
Dynamics DB	bsma.pdbj.org/dynamicsdb/
Collection of PDBj source code repositories	gitlab.com/pdbjapan/
