# Supplementary material for: Protein Data Bank Japan: Computational Resources for Analysis of Protein Structures
Source: J Mol Biol. Author manuscript; Available in PMC 2025 Dec 28. (PMC12744820; doi:10.1016/j.jmb.2025.169013)
Supplement: Supplementary file [file NIHMS2126339-supplement-Supplementary_file.pdf]

Protein Data Bank Japan: Computational resources for analysis of protein structures

Gert-Jan Bekker,[1] Chioko Nagao,[1] Matsuyuki Shirota,[2,3,4] Tsukasa Nakamura,[5,6] Toshiaki Katayama,[1,7] Daisuke Kihara,[1,5,6,8] Kengo Kinoshita,[2,3,4] Genji Kurisu [1,9]

[1] Institute for Protein Research, Osaka University, 3-2, Yamadaoka, Suita, Osaka 565-0871, Japan.

[2] Tohoku Medical Megabank Organization, Tohoku University, Sendai, Miyagi 980-8573, Japan.

[3] Advanced Research Center for Innovations in Next-Generation Medicine, Tohoku University, Sendai, Miyagi, 980-8573, Japan.

[4] Graduate School of Information Sciences, Tohoku University, Sendai, Miyagi, 980-8579, Japan.

[5] Department of Biological Sciences, Purdue University, West Lafayette, Indiana 47907, USA.

[6] Structural Biology Research Center, Institute of Material Structure Science, High Energy Accelerator Research Organization, 1-1 Oho, Tsukuba, Ibaraki 305-0801 Japan.

[7] Database Center for Life Science, Joint Support-Center for Data Science Research, Research Organization of Information and Systems, Kashiwa, Chiba 277-0871, Japan.

[8] Department of Computer Science, Purdue University, West Lafayette, Indiana, 47907, USA.

[9] Protein Research Foundation, Ina 4-1-2, Minoh, Osaka 562-8686, Japan.

Section S1: Description of simulations performed for construction of Dynamics DB data

For the selected systems, the biological unit was constructed, and neutral caps at the termini (ACE and NME) were added, if required (i.e., in case of missing residues at the termini), while missing sidechain atoms were modelled in using Chimera. A modified version of Gromacs was used to prepare the systems and perform the simulations (https://gitlab.com/gjbekker/gromacs) [1,2]. To parameterize the system, we used the Amber ff99SB-ILDN force field [3]. The first principal axis of inertia of the system was aligned with the X-axis, while the second one was with the Y-axis. A triclinic box was constructed around the system, where each residue is at least 9 Å for the box edge, and the box filled with TIP3P waters [4], and NaCl was added to neutralize the system and bring its concentration to 0.1 M, using the monovalent ion parameters by Joung et al [5]. Energy minimization was used to resolve any clashes, either in the solute or between the solute and the solvent. Next, 100 ps of NVT simulation at 300 K using the Bussi thermostat [6] with position restraints on the heavy protein atoms, with velocities initialized at 300 K following a Maxwell distribution was performed. Finally, 100 ps NPT simulation with position restraints using the Bussi barostat was used to equilibrate the pressure [7]. The long-range electrostatics were calculated using the zero-dipole summation method, which is a cutoff-based approach utilizing a well-defined pairwise function [8–10], with the damping factor α set to 0 $Å^{-1}$ and the atom-based cutoff length set to 12 Å. A time-step of 2 fs was used, with LINCS [11] to constrain the bond lengths and SETTLE [12] to constrain the water geometries.

For each system, 10 replicas were initiated from the prepared system after the above described 100ps NPT equilibration simulation, with a different random seed used to generate the velocities at 400 K. First, 100 ps at 400 K with position restraints on the heavy solute atoms was performed, followed by 100 ns at 400 K without position restraints, but with restraints on the center of mass and moment of inertia of the solute, to prevent translation and rotation within the box [1,2]. Cutoffs, electrostatics, constraints and the timestep are the same as the preparation described above. During the production run, a snapshot was saved every 10 ps for the heavy solute atoms, which were subsequently analyzed using R-value analysis [1,2].

To analyze the stability of the proteins, we used the R-value analysis. Previously, we showed that the relative stability of single chain antibodies can be estimated by calculating the average Q-value [13] (i.e. the fraction of native contacts) along MD trajectories at high temperature (400 K) [14]. Our results showed that the higher temperature MD simulations enable us to capture longer timescale dynamics, without perturbing the structures, showing a high

correlation to experimental thermal stability (Tm) values. Later, for our work related to analyzing protein-ligand complexes predicted by McMD-based dynamic docking simulations [1,2], we also introduced a new quantity, R, which calculates the fraction of contacts between two molecules with respect to a reference structure, where a value of 1.0 means that the complex forms the same interactions between these two molecules as in the reference structure [15]. For reference, the Q-value is defined as $Q = \frac{1}{N}\sum_{(i,j)} \frac{1}{1+\exp\left(\beta(r_{ij}(X) - \Lambda r_{ij}^0)\right)}$, where N is the number of native contact pairs with a distance less than 4.5 Å, $r_{ij}(X)$ the distance of the pair (i, j) in configuration X, r_{ij}^0 the distance in the experimental configuration, β a smoothing parameter and Λ a parameter to account for the fluctuations formed by the contact, set to 5.0 $Å^{-1}$ and 1.8 Å, respectively [13]. The R-value distinguishes from the Q-value in the definition of i and j and the used structure. Whereas for the Q-value i and j correspond to atoms from the whole structure, for the R-value i corresponds to atoms taken from the protein and j to atoms taken from the ligand (for our previous application in dynamic docking analysis). Finally, any reference structure's distance r_{ij}^{ref} can be used instead of the experimental structure's distance r_{ij}^0 for the R-value.

The experimental conditions of a structure differ considerably from those in the case of the structure in the MD simulation box (e.g., inside a crystal versus in solution). In addition, the approximations to physics by the force field also introduces errors. As an alternative to comparing to the experimental structure, using a structure that corresponds to the center of the MD ensemble should go a long way to eliminate those errors. The reference structure used for R-value analysis can also be calculated from the MD simulations, by calculating the average contact matrix from the MD simulations, and then taking the structure whose contact matrix is the closest to this average contact matrix to obtain a structure close to the center of the MD ensemble. Then, the R-value analysis can be used to calculate the stability of the interactions in terms of the degree of their fluctuations, providing an assessment regarding the stability of each structure, as well as the stability of individual residues.

REFERENCES

[1] G.-J. Bekker, N. Kamiya, Dynamic Docking Using Multicanonical Molecular Dynamics: Simulating Complex Formation at the Atomistic Level, in: F. Ballante (Ed.), Protein-Ligand Interactions and Drug Design, Springer US, New York, NY, 2021: pp. 187–202. https://doi.org/10.1007/978-1-0716-1209-5_11.

[2] G.-J. Bekker, N. Kamiya, Advancing the field of computational drug design using multicanonical molecular dynamics-based dynamic docking, Biophys. Rev. 14 (2022) 1349–1358. https://doi.org/10.1007/s12551-022-01010-z.

[3] K. Lindorff-Larsen, S. Piana, K. Palmo, P. Maragakis, J.L. Klepeis, R.O. Dror, D.E. Shaw, Improved Side-Chain Torsion Potentials for the Amber ff99SB Protein Force Field, Proteins 78 (2010) 1950–1958. https://doi.org/10.1002/prot.22711.

[4] W.L. Jorgensen, J. Chandrasekhar, J.D. Madura, R.W. Impey, M.L. Klein, Comparison of Simple Potential Functions for Simulating Liquid Water, J. Chem. Phys. 79 (1983) 926–935. https://doi.org/10.1063/1.445869.

[5] I.S. Joung, T.E. Cheatham, Determination of alkali and halide monovalent ion parameters for use in explicitly solvated biomolecular simulations, J. Phys. Chem. B 112 (2008) 9020–9041. https://doi.org/10.1021/jp8001614.

[6] G. Bussi, D. Donadio, M. Parrinello, Canonical Sampling Through Velocity Rescaling, J. Chem. Phys. 126 (2007) 14101. https://doi.org/10.1063/1.2408420.

[7] M. Bernetti, G. Bussi, Pressure control using stochastic cell rescaling, J. Chem. Phys. 153 (2020) 114107. https://doi.org/10.1063/5.0020514.

[8] G.-J. Bekker, N. Kamiya, M. Araki, I. Fukuda, Y. Okuno, H. Nakamura, Accurate Prediction of Complex Structure and Affinity for a Flexible Protein Receptor and Its Inhibitor, J. Chem. Theory Comput. 13 (2017) 2389–2399. https://doi.org/10.1021/acs.jctc.6b01127.

[9] I. Fukuda, Y. Yonezawa, H. Nakamura, Molecular dynamics scheme for precise estimation of electrostatic interaction via zero-dipole summation principle, J. Chem. Phys. 134 (2011) 164107. https://doi.org/10.1063/1.3582791.

[10] N. Kamiya, I. Fukuda, H. Nakamura, Application of zero-dipole summation method to molecular dynamics simulations of a membrane protein system, Chem. Phys. Lett. 568–569 (2013) 26–32. https://doi.org/10.1016/j.cplett.2013.03.014.

[11] B. Hess, P-LINCS: A Parallel Linear Constraint Solver for Molecular Simulation, J. Chem. Theory Comput. 4 (2008) 116–122. https://doi.org/10.1021/ct700200b.

[12] S. Miyamoto, P.A. Kollman, Settle - An Analytical Version of the Shake and Rattle Algorithm for Rigid Water Models, J. Comput. Chem. 13 (1992) 952–962. https://doi.org/10.1002/jcc.540130805.

[13] R.B. Best, G. Hummer, W.A. Eaton, Native contacts determine protein folding mechanisms in atomistic simulations, Proc. Natl. Acad. Sci. U.S.A. 110 (2013) 17874–17879. https://doi.org/10.1073/pnas.1311599110.

[14] G.-J. Bekker, B. Ma, N. Kamiya, Thermal stability of single-domain antibodies estimated by molecular dynamics simulations, Protein Sci. 28 (2019) 429–438. https://doi.org/10.1002/pro.3546.
[15] G.-J. Bekker, M. Araki, K. Oshima, Y. Okuno, N. Kamiya, Dynamic Docking of a Medium-Sized Molecule to Its Receptor by Multicanonical MD Simulations, J. Phys. Chem. B 123 (2019) 2479–2490. https://doi.org/10.1021/acs.jpcb.8b12419.

BSM-Arc

English 日本語

My projects Help Browse

Options

Preview the entry before release:

Preview

Copy read-only URL (e.g., for reviewers)

Notices

- Your entry "Elucidation of binding mechanism, affinity and complex structure between mWT1 tumor-associated antigen peptide and HLA-A*24:02" has been published
9/4/2023, 7:38:21 PM
- A release was requested for the entry Elucidation of binding mechanism, affinity and complex structure between mWT1 tumor-associated antigen peptide and HLA-A*24:02
9/4/2023, 7:37:03 PM

Entry editor

entry

	bsmid*	entry_doi*	RTPI	archive_group	archive_group_parent	name*	DOI*	graphical_abstract_path*	description*	deposit_date*	release_date*	modification_date*
	41	10.51093/bsm-00041				Elucidation of binding mechanism, affinity and complex structure between mWT1 tumor-associated antigen peptide and HLA-A*24:02	10.1002/pro.4775	/GA.webp		2023-03-26	2023-09-04	2023-09-04

history

	major_version*	minor_version*	release_date*	release_notes*
	1	0	2023-09-04	
	1	1		

author

	ordinal*	name	orcid	manager
×	1	Gert-Jan Bekker	0000-0001-8385-5693	true
×	2	Nobutaka Numoto	0000-0001-6113-9792	
×	3	Maki Kawasaki		
×	4	Takahiro Hayashi		
×	5	Saaya Yabuno		
×	6	Yuko Kozono		
×	7	Takeyuki Shimizu		
×	8	Haruo Kozono		
×	9	Nobutoshi Ito		
×	10	Masayuki Oda	0000-0002-8568-4223	
×	11	Narutoshi Kamiya	0000-0002-0527-6968	

All

Add author

document

	ordinal*	title	content
×	1	Abstract	We have applied our advanced computational and experimental methodologies to investigate the complex structure and binding mechanism of a modified Wilms' Tumor 1 (mWT1) protein epitope to the understudied Asian-dominant allele HLA-A*24:02 (HLA-A24). We have used our developed multicanonical molecular dynamics (McMD)-based dynamic docking method and have successfully reproduced the native complex structure, which we have also solved using X-ray crystallography. Subsequent path sampling MD simulations elucidated the atomic details of the binding process and indicated that first an encounter complex is formed between the N-terminal's positive charge and a cluster of negative residues on the surface of HLA-A24, with the HLA molecule preferring a predominantly closed state. As the peptide slowly binds, it pries open the pocket, slowly changing the HLA molecule from a closed state to an open one, where the peptide can then bind and form the native complex structure. Although the HLA molecule is in a predominately closed state, interaction with the peptide epitope can cause the pocket to open and stabilize in an open conformation with the peptide bound. The computational tools and analyses used here can also be applied to more minor HLA alleles, and could even provide useful in the development of personalized medicine, due to the ever-reducing computational requirements.

Add free-text panel

methodology

	ordinal*	type	description
×	1	methodology_Other	McMD-based dynamic docking<br>Code available at: https://gitlab.com/gjbekker/gromacs

methodology_Other

	ordinal*	DOI
×	1	10.1007/s12551-022-01010-z

Add other methodology

methodology_MD

	ordinal*	input_structure	input_restart	input_conf	input_other	output_structure	output_restart	output_other	dt	time	thermostat	barostat

Add MD settings

external_databases

	ordinal*	dbname	accession
×	1	PDB	8ISN
×	2	XRDa	122

Add link to external database

File manager

Path: /

	Name	Description	Size	Changed
	1			
	3			
	4			
	5			
	S1			
	S3			
	S4			
	data			
	movie_S1			
	GA.webp		576.63 KB	3/29/2023, 1:40:14 PM
	hla_seqs_fig_5d.fasta		36.28 KB	3/29/2023, 1:17:48 PM

Drop files/folders from your local filesystem into this panel to upload them or use the buttons below.

Select files for uploading **Select folders for uploading** **Create new folder** **Import from SFTP area**

Figure S1. Interface of BSM-Arc editor. The interface uses the same interface as that of our CIF Editor (Fig. 2). Upon creation of the entry, the title first needs to be provided, but can be modified afterwards (in *entry.name*). If any papers are available, they can be linked to via their DOI in the *entry.DOI* column (multiple can be provided, one is required for publication). Upon creation, a unique BSM-Arc ID is assigned to the entry, in addition to a DOI pointing to the entry. Authors can be added to the entry via the author category, and their ORCID ID can also be set, giving them read access to the entry, prior to release. To provide co-authors write access (for simultaneous editing or adding of files), the manager data-item can be toggled on. Using the document category, free text panels can be added to the entry for additional explanations, or, e.g., to add the abstract of the entry. Additional information that describes the methodology can also be added, as well as links to external databases. Finally, files and folders can be uploaded directly using the web-browser via drag-and-drop. Uploads are accelerated, as multiple files are uploaded simultaneously and in chunks for improved performance. Alternatively, there is an option to import files from the SFTP area, which can be uploaded either via RSYNC or via SFTP. The example uses BSM-00041 as an example. The public page of this entry is available at https://bsma.pdbj.org/entry/41.

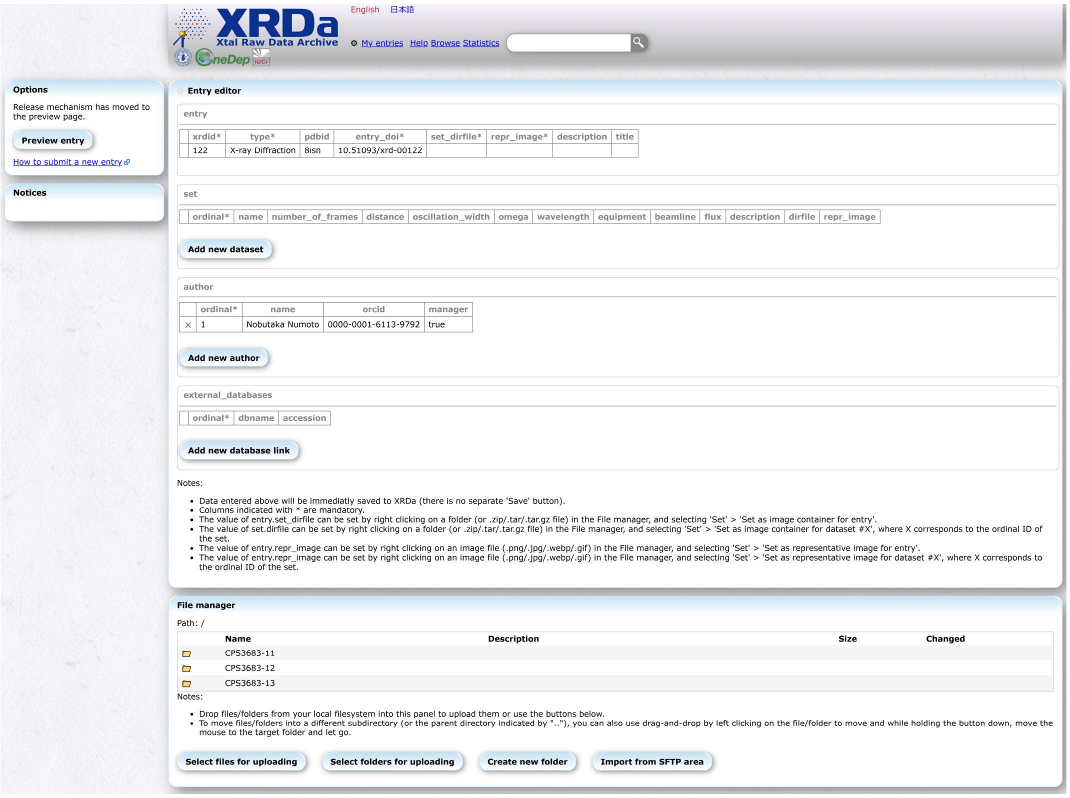

Figure S2. XRDa editor. The interface uses the same interface as that of our CIF Editor (Fig. 2). For PDB entries, much of the information provided is simply imported from the corresponding PDB entry page, but for entries not linked to a PDB entry, some information can be provided via this interface, such as the entry title and description. If any papers are available, they can be linked to via their DOI by adding them to the table in the *external_databases* category. Upon creation, a unique XRDa ID is assigned to the entry, in addition to a DOI pointing to the entry. Authors can be added to the entry via the author category, and their ORCID ID can also be set, giving them read access to the entry, prior to release. To provide co-authors write access (for simultaneous editing or adding of files), the manager data-item can be toggled on. If there are multiple data sets, these can be configured via the set category, including providing the path to the file or folder that contains the set. Finally, files and folders can be uploaded directly using the web-browser via drag-and-drop. Uploads are accelerated, as multiple files are uploaded simultaneously and in chunks for improved performance. Alternatively, there is an option to import files from the SFTP area, which can be uploaded either via RSYNC or via SFTP. The example uses XRD-00122 as an example. The public page of this entry is available at https://xrda.pdbj.org/entry/8isn.

Experimental procedure

**Experimental method**	SINGLE WAVELENGTH
**Source type**	SYNCHROTRON
**Source details**	SPRING-8 BEAMLINE BL44XU
**Synchrotron site**	SPring-8
**Beamline**	BL44XU
**Temperature [K]**	100
**Detector technology**	PIXEL
**Collection date**	2023-04-12
**Detector**	DECTRIS EIGER X 16M
**Wavelength(s)**	0.900
**Spacegroup name**	P 1 21 1
**Unit cell lengths**	66.279, 138.553, 68.947
**Unit cell angles**	90.00, 94.18, 90.00

Refinement procedure

**Resolution**	49.490 - 2.040
**R-factor**	0.1964
**Rwork**	0.195
**R-free**	0.23850
**Structure solution method**	MOLECULAR REPLACEMENT
**Starting model (for MR)**	4n58
**RMSD bond length**	0.007
**RMSD bond angle**	0.847
**Data reduction software**	XDS
**Data scaling software**	XDS
**Phasing software**	PHENIX (1.19.2_4158)
**Refinement software**	PHENIX (1.19.2_4158)

Data quality characteristics

	Overall	Outer shell
**Low resolution limit [Å]**	49.490	2.170
**High resolution limit [Å]**	2.040	2.040
**Rmerge**	0.077	0.530
**Number of reflections**	77569	23492
**<I/σ(I)>**	6.63	1.17
**Completeness [%]**	95.5	87.8
**Redundancy**	1.82	
**CC(1/2)**	0.993	0.700

Figure S3. Enhancements to the Experimental details shown in PDBj Mine. Extended listing of experimental details for crystallography derived structures. Additional data is now shown in the Mine Experimental details page, listing more information regarding the experimental procedure, the refinement procedure, and the data quality characteristics of the structure (here, the information for PDB ID 8JC1 is shown).

Sequence properties

Secondary structure parameters were calculated using S4PRED, while disorder propensities were calculated using flDPnn.

M A K K T S S K G K L P P G P R P L P L L G N L L Q M D R R G L L K S F L R F R
E K Y G D V F T V H L G P R P V V M L C G V E A I R E A L V D K A E A F S G R G
K I A M V D P F F R G Y G V V F A N G N R W K V L R R F S V T T M R D F G M G K
R S V E E R I Q E E A Q C L I E E L R K S K G A L M D P T F L F Q S I T A N I I
C S I V F G K R F H Y Q D Q E F L K M L N L F Y Q T F S L I S S V F G Q L F E L
F S G F L K H F P G A H R Q V Y K N L Q E I N A Y I G H S V E K H R E T L D P S
A P R D L I D T Y L L H M E K E K S N A H S E F S H Q N L N L N T L S L F F A G
T E T T S T T L R Y G F L L M L K Y P H V A E R V Y R E I E Q V I G P H R P P E
L H D R A K M P Y T E A V I Y E I Q R F S D L L P M G V P H I V T Q H T S F R G
Y I I P K D T E V F L I L S T A L H D P H Y F E K P D A F N P D H F L D A N G A
L K K T E A F I P F S L G K R I C L G E G I A R A E L F L F F T T I L Q N F S M
A S P V A P E D I D L T P Q E C G V G K I P P T Y Q I R F L P R H H H H

Color info:
- Coiled coil: white box; Helix: magenta box; Sheet: yellow box.
- Blue border: > 30 % chance of disordered residue; Green border: > 50 % chance of disordered residue that binds protein.
Move the mouse over the residues to view secondary structure and disorder propensities.

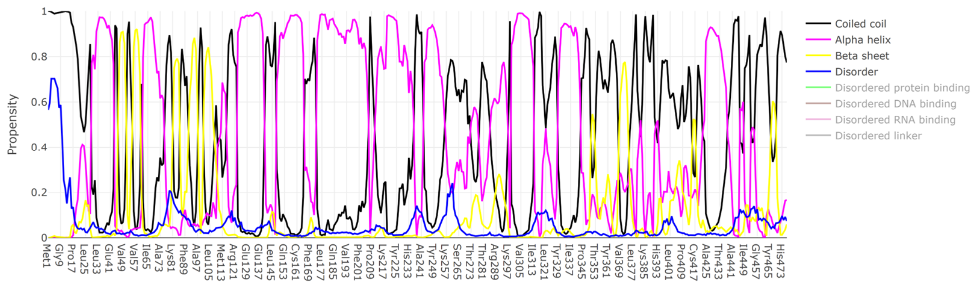

The above graph is interactive:
- Click items in the legend to toggle them.
- Mouse over the graphs to see the values.
- Left-click and drag to zoom into a specific region; double click to return.
Note that not all residues might be listed on the x-axis due to limited space, but are included in the graph (zooming will show them).

Kyte-Doolittle hydrophathy plot.

Window size: 9

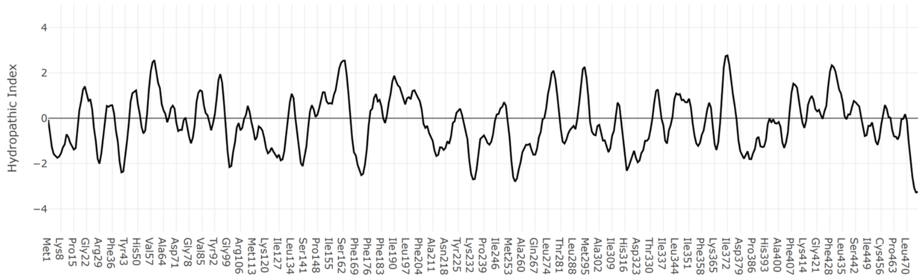

The above graph is also interactive.
Values larger than 0.0 correspond to hydrophobic regions, while values less than 0.0 correspond to hydrophilic regions.

Homology search

PDB homology	306 results were found.<br>Best score: 100% 5ufg	View detailed results
AlphaFold DB homology	10000 results were found.<br>Best score: 98% P20813	View detailed results
SwissProt homology	1418 results were found.<br>Best score: 98% P20813	View detailed results

Literature search

34 PubMed entries within for selected entries in the Summary panel.

Enter keywords to search PubMed

Figure S4. Sequence Navigator Pro lower panels. The top panel lists the predicted properties of the sequence. In the top section, the sequence is listed, with each residue colored based on the predicted secondary structure (coil: white, helix: magenta, sheet: yellow). The border of the residue box is colored based on the disordered probability (> 30% chance of disordered: blue, > 50% chance of disordered while binding to protein: green). In the middle section, the secondary structure and disordered propensities along the sequence are graphed following a similar color scheme as the sequence markup in the top widget. In the bottom section, the Kyle-Doolittle hydropathy plot is shown along the sequence. The middle panel corresponds to the homology report panel, while the bottom one corresponds to the Literature search panel. Clicking on each of the results shows the homology search results in an equivalent manner to our Sequence Navigator search results. Also shown is the Literature search panel, where the PubMed abstracts of the selected entries from the summary panel can be searched here by entering the keywords in the search field and hitting ENTER or clicking the search icon (show all matching articles by providing an empty input). The results are then shown in a new window, as shown in Fig. S6.

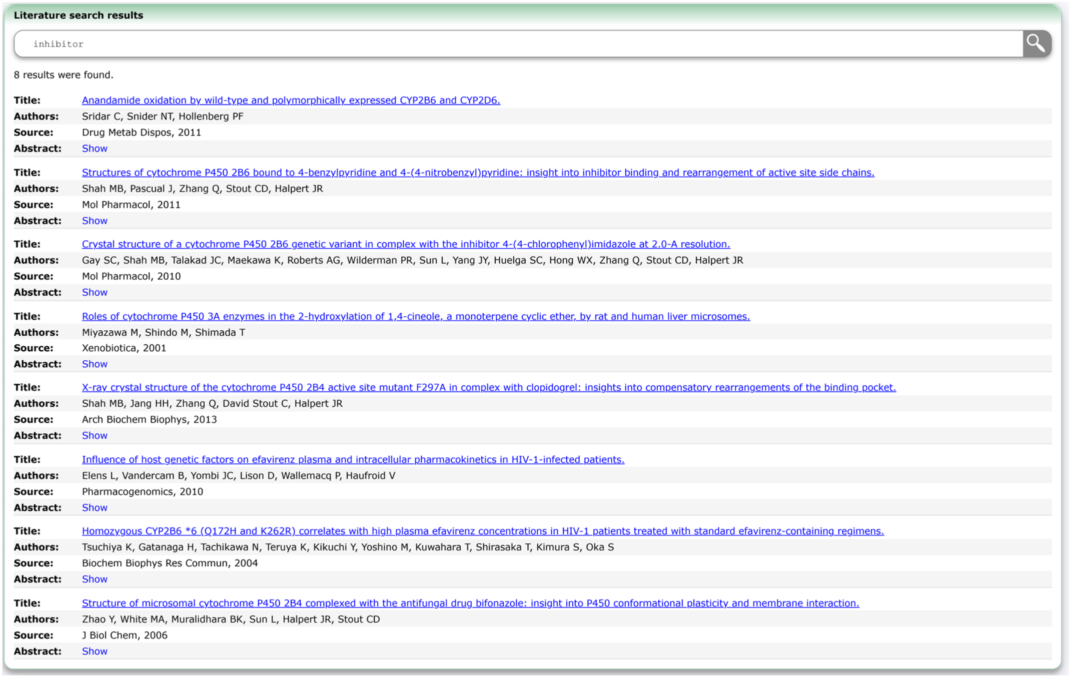

Figure S5. PubMed search results. The matching PubMed articles for the given keyword (here: “inhibitors”) are shown. Clicking on the title opens the PubMed entry, while clicking on the “Show” link for the Abstract, shows the abstract for the article.

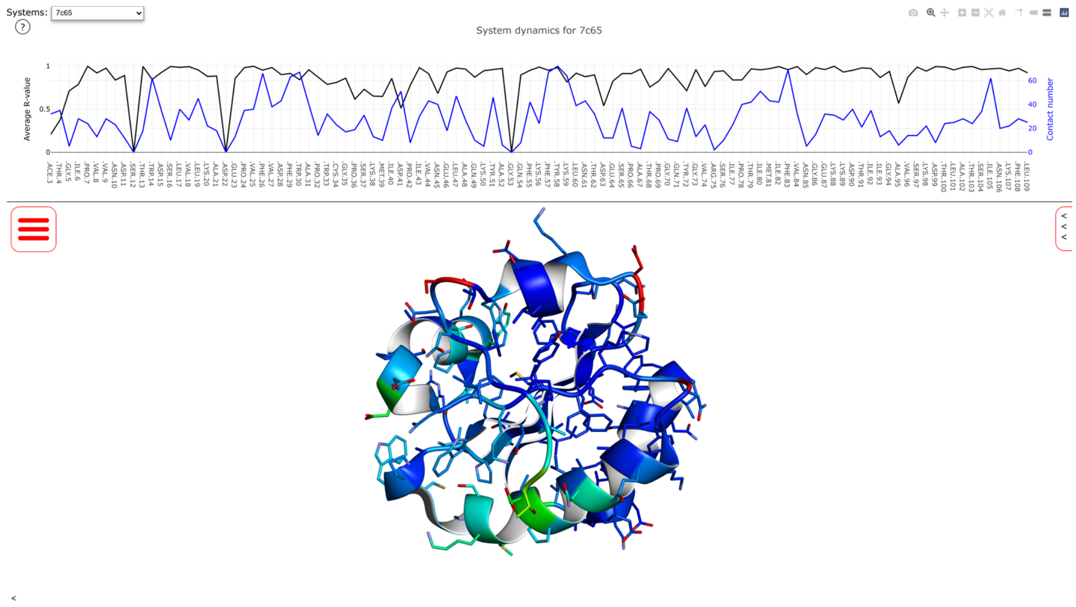

Figure S6. Example of Dynamics DB entry. In the top section, the stability of the structure along the sequence is shown in terms of the R-value (black), and the number of contacts made by each residue in the representative structure (blue). In the bottom section, the representative structure is shown and visualized by our molecular viewer Molmil, with the cartoon and carbons colored based on the stability score (R-value).
